# The crystal structures of tetra­kis­(μ-*n*-butyrato-κ^2^ *O*:*O*′)bis[bromidorhenium(III)] and tetra­kis­(μ-*n*-butyrato-κ^2^ *O*:*O*′)bis[chlorido­rhenium(III)] aceto­nitrile disolvate

**DOI:** 10.1107/S2056989015020563

**Published:** 2015-11-07

**Authors:** Carly R. Reed, William W. Brennessel

**Affiliations:** a Department of Chemistry and Biochemistry, The College at Brockport, SUNY, Brockport, NY 14420, USA; b Department of Chemistry, University of Rochester, Rochester, NY 14627, USA

**Keywords:** crystal structure, dihalido­tetra­kis­(butyrato)dirhenium(III,III), dirhenium tetra­carboxyl­ate, quadruple bond, paddlewheel complex

## Abstract

The title complexes, [Re_2_Br_2_(O_2_CC_3_H_7_)_4_], (**1**), and [Re_2_(O_2_CC_3_H_7_)_4_Cl_2_]·2CH_3_CN, (**2**), both exhibit paddlewheel structures with four carboxyl­ate ligands bridging two Re^III^ atoms. The Re—Re distances are 2.2325 (2) and 2.2299 (3) Å, indicating quadruple bonds between the Re^III^ atoms in each complex. Both complexes contain an inversion center at the mid-point of the Re—Re bond. The Re—Br bond [2.6712 (3) Å] in (**1**) is 0.1656 (6) Å longer than the Re—Cl distance [2.5056 (5) Å] of (**2**). In (**2**), the N atom of each co-crystallized aceto­nitrile solvent mol­ecule is nearly equidistant between and in close contact with two carboxyl­ate C atoms.

## Abstract 2

The structures of [Re_2_Br_2_(O_2_CC_3_H_7_)_4_] and [Re_2_(O_2_CC_3_H_7_)_4_Cl_2_]·2CH_3_CN are reported here. Both complexes, crystallized from aceto­nitrile and diethyl ether, exhibit paddlewheel conformations with quadruple bonds between the Re atoms.

## Chemical context   

The first compound discovered to contain a metal–metal quadruple bond was K_2_Re_2_Cl_8_·2H_2_O (Cotton & Harris, 1965 ▸); since then numerous other quadruply bonded complexes have been isolated (Cotton *et al.*, 2005 ▸). Dirhenium quadruply bonded complexes are of inter­est due to their ability to act as mol­ecular building blocks for the formation of mol­ecular triangles and other multiple-metal arrays in which electronic coupling and delocalization between metal sites can be explored (Bera, Angaridis *et al.* 2001 ▸; Bera, Smucker *et al.*, 2001 ▸; Vega *et al.*, 2002 ▸). The title complexes are of the structural type classified as paddlewheel complexes, where the four carboxyl­ate ligands bridge the two metal atoms, creating a paddlewheel appearance. A variety of these dirhenium(III) tetra­carboxyl­ate complexes were synthesized by Cotton *et al.* (1966 ▸) and in subsequent years the crystal structures of [Re_2_Cl_2_(O_2_CCH_3_)_4_], [Re_2_Cl_2_(O_2_CC_6_H_5_)_4_] (Bennett *et al.*, 1968 ▸), [Re_2_(ReO_4_)_2_(O_2_CC_3_H_7_)_4_] (Calvo *et al.*, 1970 ▸), [Re_2_ *X*_2_{O_2_CC(CH_3_)_3_}_4_], where *X* = Cl or Br (Collins *et al.*, 1979 ▸), [Re_2_Cl_2_(O_2_CCH_3_)_4_] (Koz’min *et al.*, 1980 ▸), and [Re_2_Cl_2_(O_2_CC_3_H_7_)_4_] (Thomson *et al.*, 2014 ▸) have been reported. For additional dirhenium tetra­carboxyl­ate structures, see: Shtemenko *et al.* (2001 ▸), Cotton *et al.* (1997 ▸), and Vega *et al.* (2002 ▸). This communication reports and compares the structures of [Re_2_Br_2_(O_2_CC_3_H_7_)_4_], (**1**), and [Re_2_(O_2_CC_3_H_7_)_4_Cl_2_]·2CH_3_CN, (**2**). 

## Structural commentary   

**Figure 1:**
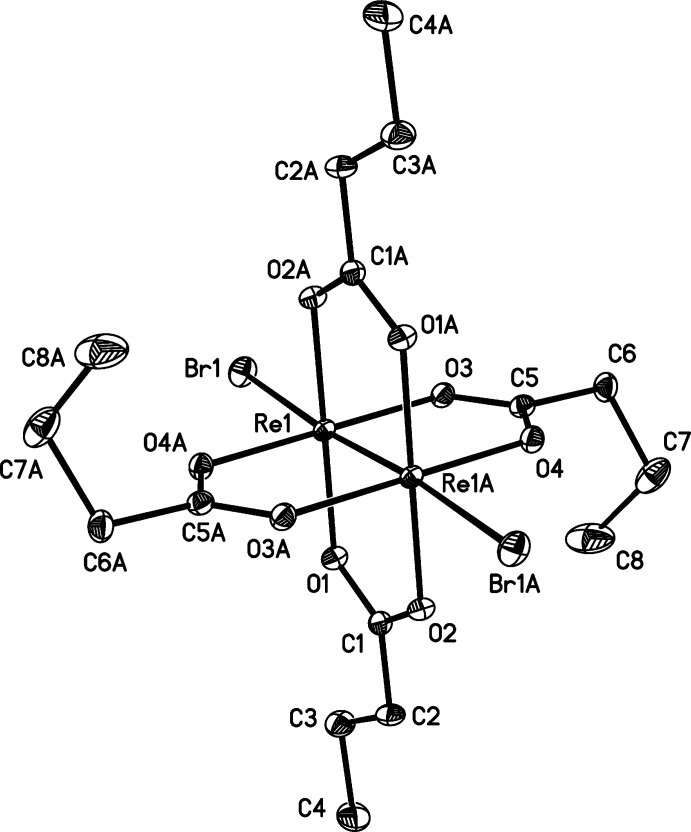
The mol­ecular structure of title compound (**1**), with displacement ellipsoids drawn at the 50% probability level. The symmetry-equivalent half is generated by operator (−*x* + 1, −*y* + 1, −*z* + 1).

Both of the title dirhenium metal complexes are located on crystallographic inversion centers that coincide with the midpoint of the Re—Re bonds. The short Re—Re bond lengths of 2.2325 (2) and 2.2299 (3) Å, in (**1**) and (**2**), respectively, are indicative of quadruple bonds (Tables 1 ▸ and 2 ▸). The four butyrate groups bridge the two Re^III^ metal atoms in both cases, forming the anti­cipated paddlewheel structures (Figs. 1 ▸ and 2 ▸).
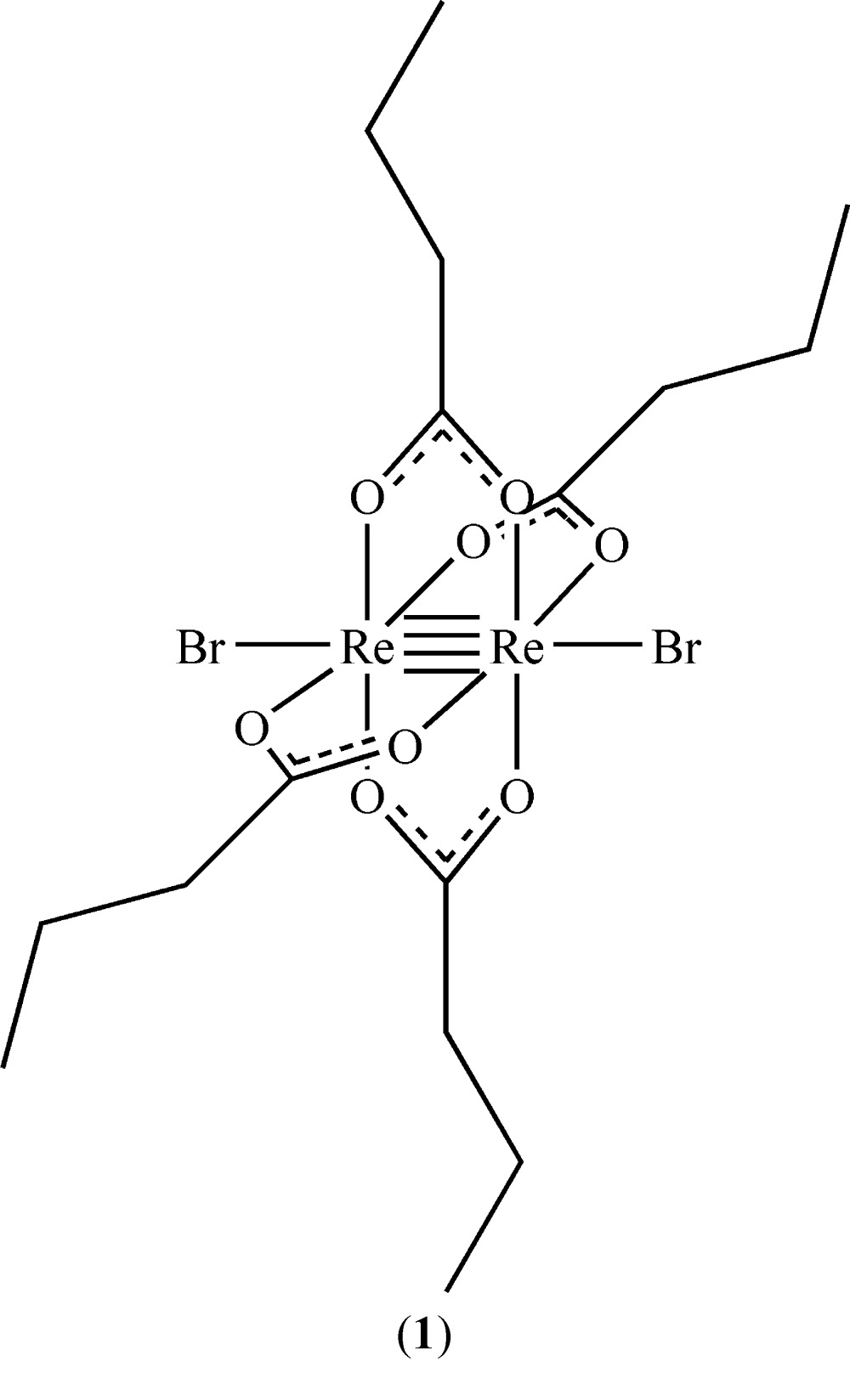
 
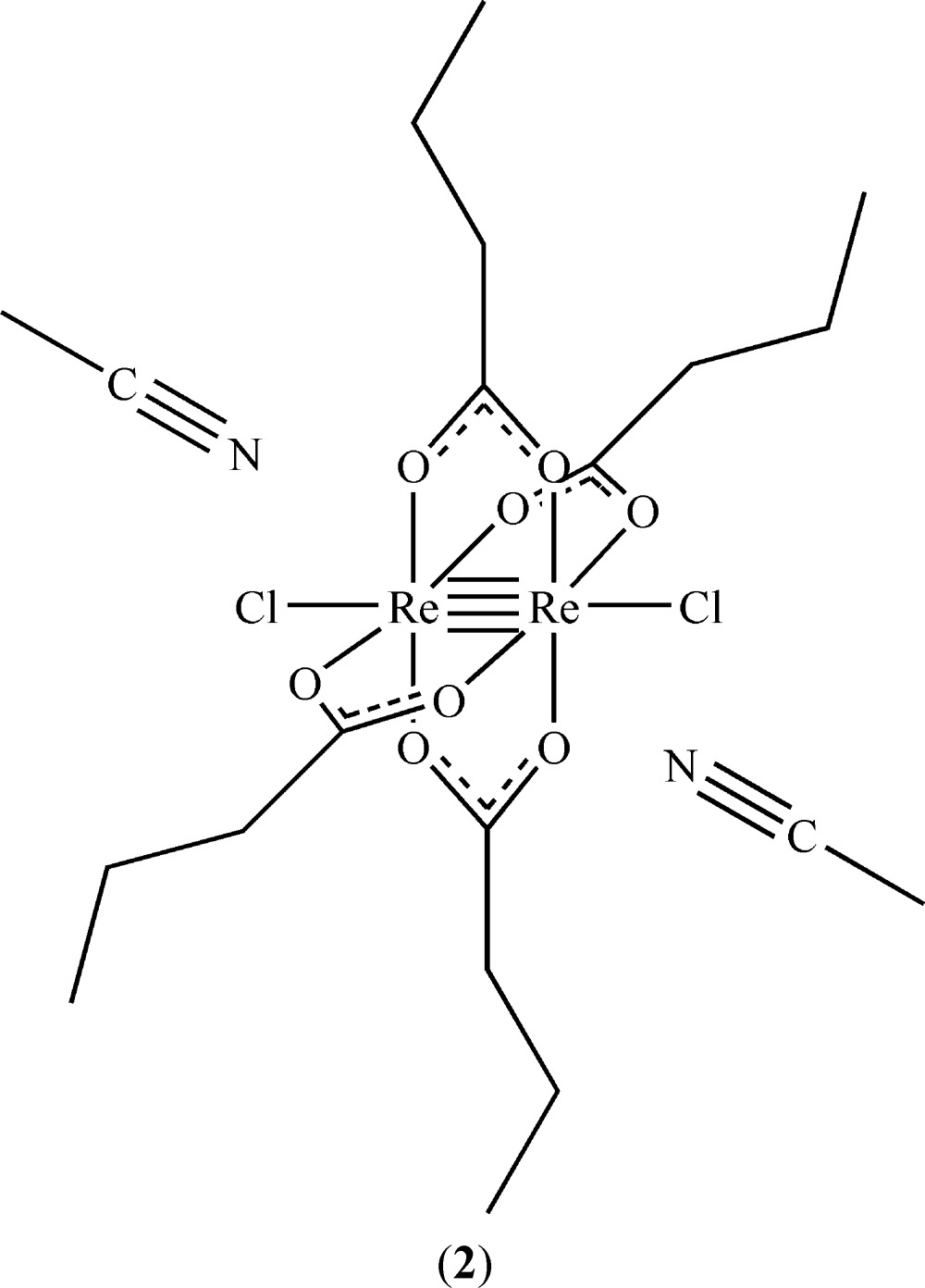


 The asymmetric unit of (**2**) also contains one co-crystallized aceto­nitrile solvent mol­ecule in a general position, thus giving rise to twice that in the formula unit.

The *X*—Re—Re—*X* bonds in (**1**) and (**2**) are nearly linear, as can be seen in the Re—Re—Br [175.018 (7)°] and Re—Re—Cl [178.254 (11)°] bond angles, and are comparable to those observed in similar compounds (Collins *et al.*, 1979 ▸; Thomson *et al.*, 2014 ▸). The Re—Cl bond length [2.5056 (5) Å] of (**2**) is similar to those of the previously published analog without co-crystallized aceto­nitrile (Thomson *et al.*, 2014 ▸), [Re_2_Cl_2_(O_2_CC(CH_3_)_3_)_4_], and [Re_2_Cl_2_(O_2_CC_6_H_5_)_4_]·2CHCl_3_ (Bennett *et al.*, 1968 ▸; Collins *et al.*, 1979 ▸). The Re—Br bond length [2.6712 (3) Å] of (**1**) is slightly longer than the Re—Br bond [2.603 (1) Å] found in [Re_2_Br_2_(O_2_CC(CH_3_)_3_)_4_] (Collins *et al.*, 1979 ▸). The Re—Br and Re—Cl distances of (**1**) and (**2**) differ by 0.1656 (6) Å and those of Cotton and coworkers differ by 0.126 (3), both of which are consistent with the difference in covalent radii of Cl and Br (0.15 Å).

The structure of (**1**) is isotypic with the chlorido analog published by Thomson *et al.* (2014 ▸). Inspection of the torsion angles of the hydro­carbon chains reveals the possible effect of the co-crystallization of solvent in [Re_2_Cl_2_(O_2_CC_3_H_7_)_4_]. In compound (**2**), the C1—C2—C3—C4 torsion angle is −70.2 (2)°, comparable to −67.9 (2)° for C5—C6—C7—C8 (Fig. 1 ▸). In the structure of [Re_2_Cl_2_(O_2_CC_3_H_7_)_4_] without co-crystallizing solvent, the torsion angles vary more [C1—C2—C3—C4 = −55.2 (5) and C5—C6—C7—C8, 179.5 (4)°] (Thomson *et al.*, 2014 ▸), similar to those observed in (**1**) (Table 1 ▸).

## Supra­molecular features   

**Figure 3:**
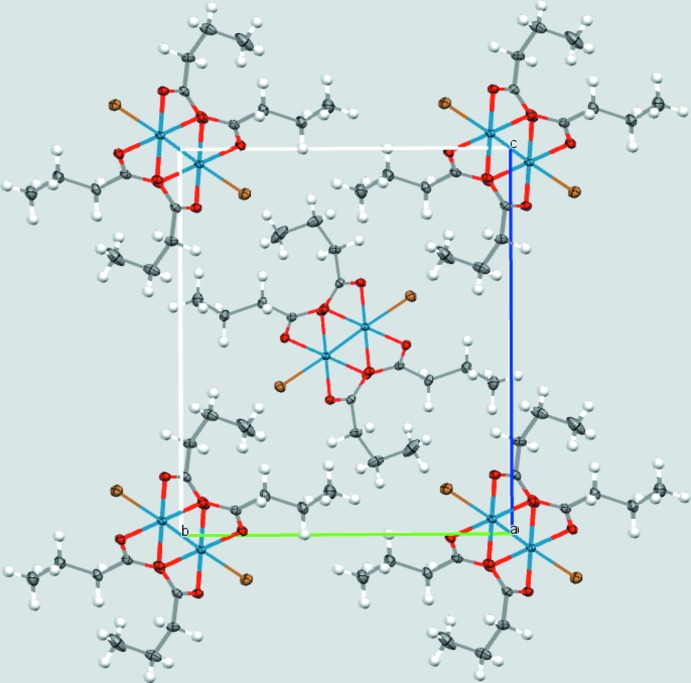
The packing arrangements of (**1**).

Packing arrangements are shown in Figs. 3 ▸ and 4 ▸. In (**2**) nitro­gen atom N1 of the co-crystallized aceto­nitrile solvent mol­ecule is located at distances of 3.197 (3) and 3.216 (3) Å from the carboxyl­ate carbon atoms C1 and C5, respectively. This is just within the sum of the van der Waals radii of 3.25 Å (Bondi, 1964 ▸), and suggests the presence of a weak electrostatic inter­action between the solvent and dirhenium species.

## Database survey   

There are 145 structures in the Cambridge Structural Database to date (CSD, Version 5.36, update No. 3, May 2015; Groom & Allen, 2014 ▸) that have explicitly defined Re—Re quadruple bonds. However, this appears to be an inconsistent denotation, as many other structures that contain quadruple bonds are not presented as such. For instance only six of the eleven carboxyl­ate paddlewheel complexes in the CSD (to date) have their Re—Re bonds defined as quadruple. Thus a better way to search appears to be by bond length. There are 298 entries with Re—Re bond lengths ≤ 2.29 Å. The only examples of defined quadruple bonds greater than this (excluding obviously disordered structures) are two dirhenium structures with bridging hydride ligands (CSD refcodes BIBLED and BIBLIH; Green *et al.*, 1982 ▸) and two with bridging di-*p*-tolyl­formamidine ligands (CSD refcodes KOZFUA and KOZGEL; Cotton & Ren, 1992 ▸).

## Synthesis and crystallization   

The title compounds were previously synthesized *via* microwave irradiation and fully characterized by elemental analysis and UV–Vis and IR spectroscopies (Reed *et al.*, 2015 ▸). For crystallization each compound was dissolved in aceto­nitrile and a few drops of diethyl ether were added to the aceto­nitrile solution which produced seed crystals. Slow evaporation of the solvent at room temperature in a glovebox produced single crystals suitable for X-ray diffraction.

## Refinement   

Crystal data, data collection and structure refinement details are summarized in Table 3 ▸. H atoms were placed geometrically and treated as riding atoms: methyl­ene, C—H = 0.99 Å, with *U*_iso_(H) = 1.2*U*_eq_(C) and methyl, C—H = 0.98 Å, with *U*_iso_(H) = 1.5*U*_eq_(C).

## Supplementary Material

Crystal structure: contains datablock(s) 1, 2, global. DOI: 10.1107/S2056989015020563/pj2025sup1.cif

Structure factors: contains datablock(s) 1. DOI: 10.1107/S2056989015020563/pj20251sup2.hkl

Structure factors: contains datablock(s) 2. DOI: 10.1107/S2056989015020563/pj20252sup3.hkl

CCDC references: 1434257, 1434256

Additional supporting information:  crystallographic information; 3D view; checkCIF report

## Figures and Tables

**Figure 2:**
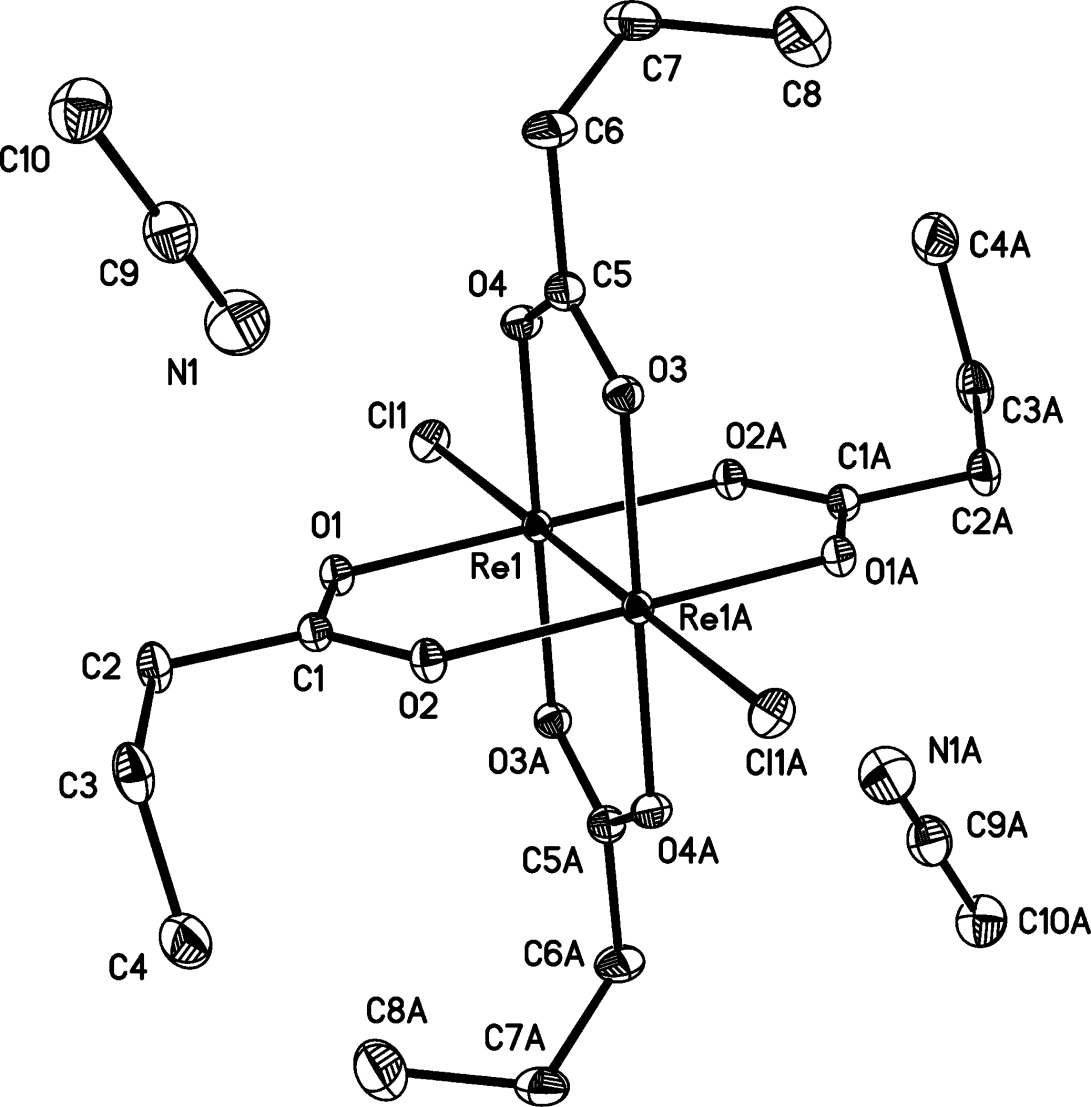
The mol­ecular structure of title compound (**2**), with displacement ellipsoids drawn at the 50% probability level. The symmetry-equivalent half is generated by operator (−*x*, −*y*, −*z* + 1).

**Figure 4:**
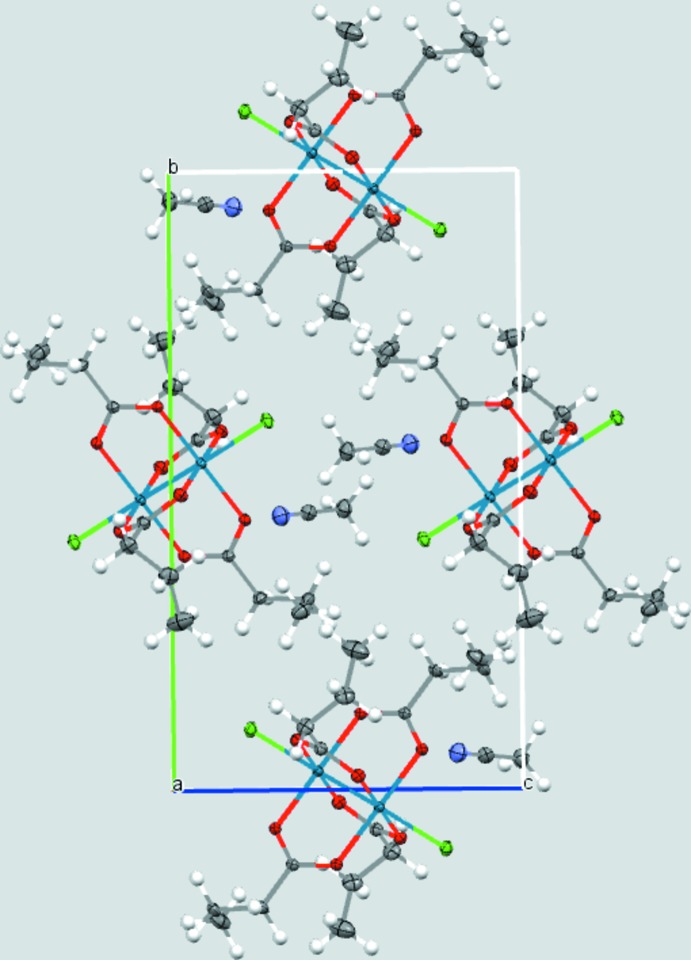
The packing arrangements of (**2**).

**Table 1:** Selected geometric parameters (Å, °) for (**1**)

Re1—O4^i^	2.0102 (15)	Re1—O3	2.0295 (14)
Re1—O2^i^	2.0159 (14)	Re1—Re1^i^	2.2325 (2)
Re1—O1	2.0225 (15)	Re1—Br1	2.6712 (3)
			
O4^i^—Re1—O2^i^	89.22 (6)	O1—Re1—O3	89.64 (6)
O4^i^—Re1—O1	90.42 (6)	Re1^i^—Re1—Br1	175.018 (7)
O2^i^—Re1—O3	90.72 (6)		
			
C1—C2—C3—C4	−177.54 (19)	C5—C6—C7—C8	56.0 (3)

Symmetry code: (i) 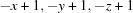.

**Table 2:** Selected geometric parameters (Å, °) for (**2**)

Re1—O1	2.0216 (12)	Re1—O4	2.0255 (12)
Re1—O2^i^	2.0217 (12)	Re1—Re1^i^	2.2299 (3)
Re1—O3^i^	2.0238 (12)	Re1—Cl1	2.5056 (5)
			
O1—Re1—O3^i^	89.75 (5)	O2^i^—Re1—O4	89.75 (5)
O2^i^—Re1—O3^i^	90.13 (5)	Re1^i^—Re1—Cl1	178.254 (11)
O1—Re1—O4	90.37 (5)		
			
C1—C2—C3—C4	−70.2 (2)	C5—C6—C7—C8	−67.9 (2)

Symmetry code: (i) 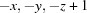.

**Table 3:** Experimental details

	(**1**)	(**2**)
Crystal data		
Chemical formula	[Re_2_Br_2_(C_4_H_7_O_2_)_4_]	[Re_2_(C_4_H_7_O_2_)_4_Cl_2_]·2C_2_H_3_N
*M*_r_	880.60	873.79
Crystal system, space group	Monoclinic, *P*2_1_/*n*	Monoclinic, *P*2_1_/*c*
Temperature (K)	100	100
*a*, *b*, *c* (Å)	6.6833 (5), 12.2817 (10), 14.6134 (12)	8.5589 (13), 17.097 (3), 10.0494 (15)
β (°)	100.5380 (16)	105.830 (3)
*V* (Å^3^)	1179.27 (16)	1414.8 (4)
*Z*	2	2
Radiation type	Mo *K*α	Mo *K*α
μ (mm^−1^)	13.68	8.78
Crystal size (mm)	0.36 × 0.16 × 0.12	0.36 × 0.34 × 0.12

Data collection		
Diffractometer	Bruker SMART APEXII CCD platform	Bruker *SMART* APEXII CCD platform
Absorption correction	Multi-scan (*SADABS*; Sheldrick, 2014 ▸)	Multi-scan (*SADABS*; Sheldrick, 2014 ▸)
*T*_min_, *T*_max_	0.161, 0.440	0.187, 0.440
No. of measured, independent and observed [*I* > 2σ(*I*)] reflections	43018, 6464, 5503	51215, 7730, 6874
*R*_int_	0.039	0.038
(sin θ/λ)_max_ (Å^−1^)	0.875	0.876

Refinement		
*R*[*F* ^2^ > 2σ(*F* ^2^)], *wR*(*F* ^2^), *S*	0.021, 0.047, 1.04	0.021, 0.040, 1.15
No. of reflections	6464	7730
No. of parameters	129	157
H-atom treatment	H-atom parameters constrained	H-atom parameters constrained
Δρ_max_, Δρ_min_ (e Å^−3^)	2.11, −1.56	1.42, −1.61

Computer programs: *APEX2* and *SAINT* (Bruker, 2014 ▸), *SIR2011* (Burla *et al.*, 2012 ▸), *SHELXL2014* (Sheldrick, 2015 ▸) and *SHELXTL* (Sheldrick, 2008 ▸).

## supplementary crystallographic information

### (1) Tetrakis(µ-n-butyrato-κ2O:O')bis[bromidorhenium(III)] Crystal data

**Table:**

[Re_2_Br_2_(C_4_H_7_O_2_)_4_]	*F*(000) = 816
*M**_r_* = 880.60	*D*_x_ = 2.480 Mg m^−^^3^
Monoclinic, *P*2_1_/*n*	Mo *K*α radiation, λ = 0.71073 Å
*a* = 6.6833 (5) Å	Cell parameters from 3849 reflections
*b* = 12.2817 (10) Å	θ = 3.3–38.2°
*c* = 14.6134 (12) Å	µ = 13.68 mm^−^^1^
β = 100.5380 (16)°	*T* = 100 K
*V* = 1179.27 (16) Å^3^	Needle, orange
*Z* = 2	0.36 × 0.16 × 0.12 mm

### (1) Tetrakis(µ-n-butyrato-κ2O:O')bis[bromidorhenium(III)] Data collection

**Table:**

Bruker SMART APEXII CCD platform diffractometer	5503 reflections with *I* > 2σ(*I*)
Radiation source: fine-focus sealed tube	*R*_int_ = 0.039
ω scans	θ_max_ = 38.5°, θ_min_ = 2.2°
Absorption correction: multi-scan (SADABS; Sheldrick, 2014)	*h* = −11→11
*T*_min_ = 0.161, *T*_max_ = 0.440	*k* = −21→21
43018 measured reflections	*l* = −25→25
6464 independent reflections	

### (1) Tetrakis(µ-n-butyrato-κ2O:O')bis[bromidorhenium(III)] Refinement

**Table:**

Refinement on *F*^2^	Primary atom site location: structure-invariant direct methods
Least-squares matrix: full	Secondary atom site location: difference Fourier map
*R*[*F*^2^ > 2σ(*F*^2^)] = 0.021	Hydrogen site location: inferred from neighbouring sites
*wR*(*F*^2^) = 0.047	H-atom parameters constrained
*S* = 1.04	*w* = 1/[σ^2^(*F*_o_^2^) + (0.0202*P*)^2^ + 0.9723*P*] where *P* = (*F*_o_^2^ + 2*F*_c_^2^)/3
6464 reflections	(Δ/σ)_max_ = 0.001
129 parameters	Δρ_max_ = 2.11 e Å^−^^3^
0 restraints	Δρ_min_ = −1.56 e Å^−^^3^

### (1) Tetrakis(µ-n-butyrato-κ2O:O')bis[bromidorhenium(III)] Special details

**Table:**

Geometry. All e.s.d.'s (except the e.s.d. in the dihedral angle between two l.s. planes) are estimated using the full covariance matrix. The cell e.s.d.'s are taken into account individually in the estimation of e.s.d.'s in distances, angles and torsion angles; correlations between e.s.d.'s in cell parameters are only used when they are defined by crystal symmetry. An approximate (isotropic) treatment of cell e.s.d.'s is used for estimating e.s.d.'s involving l.s. planes.

### (1) Tetrakis(µ-n-butyrato-κ2O:O')bis[bromidorhenium(III)] Fractional atomic coordinates and isotropic or equivalent isotropic displacement parameters (Å^2^)

**Table:**

	*x*	*y*	*z*	*U*_iso_*/*U*_eq_	
Re1	0.41766 (2)	0.44073 (2)	0.53764 (2)	0.00983 (2)	
Br1	0.19163 (3)	0.30257 (2)	0.61673 (2)	0.01786 (4)	
O1	0.5665 (2)	0.31578 (12)	0.48971 (10)	0.0133 (2)	
O2	0.7293 (2)	0.43464 (12)	0.41390 (10)	0.0128 (2)	
O3	0.1966 (2)	0.43002 (12)	0.42259 (10)	0.0130 (2)	
O4	0.3633 (2)	0.54841 (12)	0.34843 (10)	0.0131 (2)	
C1	0.6954 (3)	0.33636 (16)	0.43620 (13)	0.0122 (3)	
C2	0.8048 (3)	0.24669 (16)	0.39774 (14)	0.0146 (3)	
H2A	0.9528	0.2611	0.4142	0.018*	
H2B	0.7672	0.2486	0.3290	0.018*	
C3	0.7644 (4)	0.13262 (17)	0.43061 (16)	0.0182 (4)	
H3A	0.6184	0.1147	0.4112	0.022*	
H3B	0.7982	0.1293	0.4994	0.022*	
C4	0.8936 (4)	0.05015 (19)	0.38890 (18)	0.0239 (5)	
H4A	0.8633	−0.0234	0.4084	0.036*	
H4B	1.0381	0.0661	0.4107	0.036*	
H4C	0.8622	0.0549	0.3208	0.036*	
C5	0.2113 (3)	0.48493 (16)	0.34960 (13)	0.0124 (3)	
C6	0.0567 (3)	0.47160 (19)	0.26301 (14)	0.0165 (4)	
H6A	0.0077	0.5441	0.2391	0.020*	
H6B	−0.0611	0.4302	0.2771	0.020*	
C7	0.1485 (4)	0.4111 (2)	0.18869 (17)	0.0251 (5)	
H7A	0.0414	0.3999	0.1330	0.030*	
H7B	0.2565	0.4566	0.1701	0.030*	
C8	0.2376 (4)	0.3019 (3)	0.2226 (2)	0.0373 (7)	
H8A	0.2849	0.2640	0.1715	0.056*	
H8B	0.1331	0.2579	0.2443	0.056*	
H8C	0.3524	0.3130	0.2740	0.056*	

### (1) Tetrakis(µ-n-butyrato-κ2O:O')bis[bromidorhenium(III)] Atomic displacement parameters (Å^2^)

**Table:**

	*U*^11^	*U*^22^	*U*^33^	*U*^12^	*U*^13^	*U*^23^
Re1	0.01032 (3)	0.01015 (3)	0.00965 (3)	−0.00067 (2)	0.00351 (2)	−0.00002 (2)
Br1	0.01861 (9)	0.01864 (9)	0.01763 (9)	−0.00465 (7)	0.00674 (7)	0.00246 (7)
O1	0.0140 (6)	0.0128 (6)	0.0137 (6)	0.0002 (5)	0.0045 (5)	−0.0012 (5)
O2	0.0124 (6)	0.0132 (6)	0.0139 (6)	0.0008 (5)	0.0050 (5)	−0.0011 (5)
O3	0.0125 (6)	0.0153 (6)	0.0115 (6)	−0.0009 (5)	0.0029 (4)	−0.0002 (5)
O4	0.0140 (6)	0.0141 (6)	0.0115 (6)	−0.0006 (5)	0.0028 (5)	0.0014 (5)
C1	0.0113 (7)	0.0139 (8)	0.0112 (7)	0.0004 (6)	0.0018 (6)	−0.0007 (6)
C2	0.0143 (8)	0.0129 (8)	0.0174 (8)	0.0029 (6)	0.0049 (7)	−0.0030 (6)
C3	0.0219 (10)	0.0139 (8)	0.0198 (9)	0.0030 (7)	0.0060 (7)	−0.0017 (7)
C4	0.0274 (12)	0.0185 (10)	0.0258 (11)	0.0081 (8)	0.0048 (9)	−0.0023 (8)
C5	0.0122 (8)	0.0126 (8)	0.0125 (7)	0.0002 (6)	0.0030 (6)	−0.0020 (6)
C6	0.0162 (9)	0.0210 (9)	0.0114 (8)	−0.0008 (7)	0.0002 (6)	−0.0004 (7)
C7	0.0222 (11)	0.0361 (13)	0.0183 (9)	−0.0110 (10)	0.0068 (8)	−0.0097 (9)
C8	0.0262 (13)	0.0418 (17)	0.0430 (16)	0.0033 (12)	0.0035 (11)	−0.0264 (13)

### (1) Tetrakis(µ-n-butyrato-κ2O:O')bis[bromidorhenium(III)] Geometric parameters (Å, º)

**Table:**

Re1—O4^i^	2.0102 (15)	C3—C4	1.528 (3)
Re1—O2^i^	2.0159 (14)	C3—H3A	0.9900
Re1—O1	2.0225 (15)	C3—H3B	0.9900
Re1—O3	2.0295 (14)	C4—H4A	0.9800
Re1—Re1^i^	2.2325 (2)	C4—H4B	0.9800
Re1—Br1	2.6712 (3)	C4—H4C	0.9800
O1—C1	1.290 (2)	C5—C6	1.489 (3)
O2—C1	1.281 (2)	C6—C7	1.533 (3)
O2—Re1^i^	2.0159 (14)	C6—H6A	0.9900
O3—C5	1.281 (2)	C6—H6B	0.9900
O4—C5	1.283 (2)	C7—C8	1.514 (4)
O4—Re1^i^	2.0102 (14)	C7—H7A	0.9900
C1—C2	1.488 (3)	C7—H7B	0.9900
C2—C3	1.521 (3)	C8—H8A	0.9800
C2—H2A	0.9900	C8—H8B	0.9800
C2—H2B	0.9900	C8—H8C	0.9800
			
O4^i^—Re1—O2^i^	89.22 (6)	C2—C3—H3B	109.7
O4^i^—Re1—O1	90.42 (6)	C4—C3—H3B	109.7
O2^i^—Re1—O1	179.64 (6)	H3A—C3—H3B	108.2
O4^i^—Re1—O3	179.91 (6)	C3—C4—H4A	109.5
O2^i^—Re1—O3	90.72 (6)	C3—C4—H4B	109.5
O1—Re1—O3	89.64 (6)	H4A—C4—H4B	109.5
O4^i^—Re1—Re1^i^	90.86 (4)	C3—C4—H4C	109.5
O2^i^—Re1—Re1^i^	89.64 (4)	H4A—C4—H4C	109.5
O1—Re1—Re1^i^	90.35 (4)	H4B—C4—H4C	109.5
O3—Re1—Re1^i^	89.08 (4)	O3—C5—O4	120.85 (18)
O4^i^—Re1—Br1	93.87 (4)	O3—C5—C6	120.16 (18)
O2^i^—Re1—Br1	88.85 (4)	O4—C5—C6	118.92 (18)
O1—Re1—Br1	91.19 (4)	C5—C6—C7	110.48 (19)
O3—Re1—Br1	86.19 (4)	C5—C6—H6A	109.6
Re1^i^—Re1—Br1	175.018 (7)	C7—C6—H6A	109.6
C1—O1—Re1	119.12 (13)	C5—C6—H6B	109.6
C1—O2—Re1^i^	120.39 (13)	C7—C6—H6B	109.6
C5—O3—Re1	120.03 (13)	H6A—C6—H6B	108.1
C5—O4—Re1^i^	119.18 (13)	C8—C7—C6	112.4 (2)
O2—C1—O1	120.51 (18)	C8—C7—H7A	109.1
O2—C1—C2	118.63 (18)	C6—C7—H7A	109.1
O1—C1—C2	120.85 (18)	C8—C7—H7B	109.1
C1—C2—C3	115.75 (18)	C6—C7—H7B	109.1
C1—C2—H2A	108.3	H7A—C7—H7B	107.8
C3—C2—H2A	108.3	C7—C8—H8A	109.5
C1—C2—H2B	108.3	C7—C8—H8B	109.5
C3—C2—H2B	108.3	H8A—C8—H8B	109.5
H2A—C2—H2B	107.4	C7—C8—H8C	109.5
C2—C3—C4	109.79 (19)	H8A—C8—H8C	109.5
C2—C3—H3A	109.7	H8B—C8—H8C	109.5
C4—C3—H3A	109.7		
			
Re1^i^—O2—C1—O1	0.2 (2)	Re1—O3—C5—O4	−1.1 (3)
Re1^i^—O2—C1—C2	179.10 (13)	Re1—O3—C5—C6	175.86 (14)
Re1—O1—C1—O2	0.2 (2)	Re1^i^—O4—C5—O3	1.0 (3)
Re1—O1—C1—C2	−178.66 (14)	Re1^i^—O4—C5—C6	−175.98 (14)
O2—C1—C2—C3	177.34 (18)	O3—C5—C6—C7	−108.8 (2)
O1—C1—C2—C3	−3.7 (3)	O4—C5—C6—C7	68.2 (3)
C1—C2—C3—C4	−177.54 (19)	C5—C6—C7—C8	56.0 (3)

Symmetry code: (i) −*x*+1, −*y*+1, −*z*+1.

### (2) Tetrakis(µ-n-butyrato-κ2O:O')bis[chloridorhenium(III)] Crystal data

**Table:**

[Re_2_(C_4_H_7_O_2_)_4_Cl_2_]·2C_2_H_3_N	*F*(000) = 832
*M**_r_* = 873.79	*D*_x_ = 2.051 Mg m^−^^3^
Monoclinic, *P*2_1_/*c*	Mo *K*α radiation, λ = 0.71073 Å
*a* = 8.5589 (13) Å	Cell parameters from 3912 reflections
*b* = 17.097 (3) Å	θ = 2.4–38.3°
*c* = 10.0494 (15) Å	µ = 8.78 mm^−^^1^
β = 105.830 (3)°	*T* = 100 K
*V* = 1414.8 (4) Å^3^	Plate, orange
*Z* = 2	0.36 × 0.34 × 0.12 mm

### (2) Tetrakis(µ-n-butyrato-κ2O:O')bis[chloridorhenium(III)] Data collection

**Table:**

Bruker SMART APEXII CCD platform diffractometer	6874 reflections with *I* > 2σ(*I*)
Radiation source: fine-focus sealed tube	*R*_int_ = 0.038
ω scans	θ_max_ = 38.5°, θ_min_ = 2.4°
Absorption correction: multi-scan (SADABS; Sheldrick, 2014)	*h* = −14→14
*T*_min_ = 0.187, *T*_max_ = 0.440	*k* = −29→29
51215 measured reflections	*l* = −17→17
7730 independent reflections	

### (2) Tetrakis(µ-n-butyrato-κ2O:O')bis[chloridorhenium(III)] Refinement

**Table:**

Refinement on *F*^2^	Primary atom site location: structure-invariant direct methods
Least-squares matrix: full	Secondary atom site location: difference Fourier map
*R*[*F*^2^ > 2σ(*F*^2^)] = 0.021	Hydrogen site location: inferred from neighbouring sites
*wR*(*F*^2^) = 0.040	H-atom parameters constrained
*S* = 1.15	*w* = 1/[σ^2^(*F*_o_^2^) + (0.0071*P*)^2^ + 1.4405*P*] where *P* = (*F*_o_^2^ + 2*F*_c_^2^)/3
7730 reflections	(Δ/σ)_max_ = 0.002
157 parameters	Δρ_max_ = 1.42 e Å^−^^3^
0 restraints	Δρ_min_ = −1.61 e Å^−^^3^

### (2) Tetrakis(µ-n-butyrato-κ2O:O')bis[chloridorhenium(III)] Special details

**Table:**

Geometry. All e.s.d.'s (except the e.s.d. in the dihedral angle between two l.s. planes) are estimated using the full covariance matrix. The cell e.s.d.'s are taken into account individually in the estimation of e.s.d.'s in distances, angles and torsion angles; correlations between e.s.d.'s in cell parameters are only used when they are defined by crystal symmetry. An approximate (isotropic) treatment of cell e.s.d.'s is used for estimating e.s.d.'s involving l.s. planes.

### (2) Tetrakis(µ-n-butyrato-κ2O:O')bis[chloridorhenium(III)] Fractional atomic coordinates and isotropic or equivalent isotropic displacement parameters (Å^2^)

**Table:**

	*x*	*y*	*z*	*U*_iso_*/*U*_eq_	
Re1	0.03406 (2)	0.02939 (2)	0.41289 (2)	0.00845 (2)	
Cl1	0.11816 (5)	0.09708 (2)	0.22230 (4)	0.01465 (7)	
O1	0.11825 (15)	0.12189 (7)	0.53706 (12)	0.0114 (2)	
O2	0.05072 (15)	0.06310 (7)	0.71113 (12)	0.0116 (2)	
O3	0.18769 (15)	−0.08019 (7)	0.64715 (13)	0.0119 (2)	
O4	0.25576 (15)	−0.02169 (7)	0.47284 (13)	0.0125 (2)	
C1	0.1121 (2)	0.12144 (9)	0.66290 (17)	0.0110 (3)	
C2	0.1786 (2)	0.18996 (10)	0.75268 (19)	0.0155 (3)	
H2A	0.1391	0.2384	0.7004	0.019*	
H2B	0.2984	0.1894	0.7726	0.019*	
C3	0.1342 (3)	0.19286 (11)	0.88930 (19)	0.0190 (3)	
H3A	0.1606	0.1419	0.9367	0.023*	
H3B	0.2011	0.2333	0.9491	0.023*	
C4	−0.0440 (3)	0.21089 (13)	0.8716 (3)	0.0272 (4)	
H4A	−0.0650	0.2134	0.9626	0.041*	
H4B	−0.1109	0.1697	0.8162	0.041*	
H4C	−0.0711	0.2613	0.8244	0.041*	
C5	0.2915 (2)	−0.06622 (10)	0.57910 (18)	0.0128 (3)	
C6	0.4563 (2)	−0.10219 (12)	0.6230 (2)	0.0185 (3)	
H6A	0.4641	−0.1352	0.7055	0.022*	
H6B	0.5385	−0.0602	0.6497	0.022*	
C7	0.4947 (2)	−0.15213 (12)	0.5097 (2)	0.0226 (4)	
H7A	0.4768	−0.1204	0.4245	0.027*	
H7B	0.6109	−0.1670	0.5391	0.027*	
C8	0.3925 (3)	−0.22572 (15)	0.4773 (3)	0.0392 (6)	
H8A	0.4236	−0.2555	0.4053	0.059*	
H8B	0.2775	−0.2114	0.4447	0.059*	
H8C	0.4104	−0.2577	0.5610	0.059*	
N1	0.4670 (3)	0.05726 (13)	0.8138 (2)	0.0299 (4)	
C9	0.5906 (3)	0.05264 (13)	0.8933 (2)	0.0221 (4)	
C10	0.7485 (3)	0.04674 (14)	0.9950 (2)	0.0255 (4)	
H10A	0.7727	0.0959	1.0466	0.038*	
H10B	0.8324	0.0365	0.9475	0.038*	
H10C	0.7465	0.0038	1.0591	0.038*	

### (2) Tetrakis(µ-n-butyrato-κ2O:O')bis[chloridorhenium(III)] Atomic displacement parameters (Å^2^)

**Table:**

	*U*^11^	*U*^22^	*U*^33^	*U*^12^	*U*^13^	*U*^23^
Re1	0.00949 (2)	0.00778 (2)	0.00844 (2)	−0.00046 (2)	0.00305 (2)	0.00032 (2)
Cl1	0.01616 (16)	0.01644 (16)	0.01207 (15)	−0.00189 (13)	0.00506 (13)	0.00287 (13)
O1	0.0142 (5)	0.0097 (5)	0.0105 (5)	−0.0014 (4)	0.0037 (4)	−0.0004 (4)
O2	0.0149 (5)	0.0103 (5)	0.0097 (5)	−0.0016 (4)	0.0035 (4)	−0.0003 (4)
O3	0.0118 (5)	0.0115 (5)	0.0122 (5)	0.0009 (4)	0.0032 (4)	0.0012 (4)
O4	0.0112 (5)	0.0129 (5)	0.0140 (5)	0.0013 (4)	0.0048 (4)	0.0016 (4)
C1	0.0118 (6)	0.0094 (6)	0.0117 (6)	−0.0007 (5)	0.0031 (5)	−0.0006 (5)
C2	0.0198 (8)	0.0115 (6)	0.0151 (7)	−0.0042 (6)	0.0049 (6)	−0.0036 (6)
C3	0.0290 (9)	0.0148 (7)	0.0136 (7)	−0.0054 (7)	0.0067 (7)	−0.0046 (6)
C4	0.0316 (11)	0.0234 (9)	0.0325 (11)	−0.0057 (8)	0.0185 (9)	−0.0097 (8)
C5	0.0118 (6)	0.0123 (6)	0.0136 (7)	0.0008 (5)	0.0022 (5)	0.0002 (5)
C6	0.0114 (7)	0.0213 (8)	0.0217 (8)	0.0047 (6)	0.0027 (6)	0.0018 (7)
C7	0.0172 (8)	0.0225 (9)	0.0296 (10)	0.0056 (7)	0.0092 (7)	0.0004 (8)
C8	0.0315 (12)	0.0256 (11)	0.0572 (18)	0.0014 (9)	0.0066 (12)	−0.0116 (11)
N1	0.0290 (10)	0.0304 (10)	0.0270 (9)	0.0018 (8)	0.0018 (8)	0.0006 (8)
C9	0.0257 (9)	0.0199 (8)	0.0200 (8)	−0.0010 (7)	0.0050 (7)	0.0002 (7)
C10	0.0235 (9)	0.0288 (10)	0.0210 (9)	−0.0006 (8)	0.0005 (8)	0.0001 (8)

### (2) Tetrakis(µ-n-butyrato-κ2O:O')bis[chloridorhenium(III)] Geometric parameters (Å, º)

**Table:**

Re1—O1	2.0216 (12)	C4—H4A	0.9800
Re1—O2^i^	2.0217 (12)	C4—H4B	0.9800
Re1—O3^i^	2.0238 (12)	C4—H4C	0.9800
Re1—O4	2.0255 (12)	C5—C6	1.490 (2)
Re1—Re1^i^	2.2299 (3)	C6—C7	1.529 (3)
Re1—Cl1	2.5056 (5)	C6—H6A	0.9900
O1—C1	1.280 (2)	C6—H6B	0.9900
O2—C1	1.282 (2)	C7—C8	1.516 (3)
O2—Re1^i^	2.0217 (12)	C7—H7A	0.9900
O3—C5	1.283 (2)	C7—H7B	0.9900
O3—Re1^i^	2.0238 (12)	C8—H8A	0.9800
O4—C5	1.279 (2)	C8—H8B	0.9800
C1—C2	1.493 (2)	C8—H8C	0.9800
C2—C3	1.522 (3)	N1—C9	1.141 (3)
C2—H2A	0.9900	C9—C10	1.459 (3)
C2—H2B	0.9900	C10—H10A	0.9800
C3—C4	1.518 (3)	C10—H10B	0.9800
C3—H3A	0.9900	C10—H10C	0.9800
C3—H3B	0.9900		
			
O1—Re1—O2^i^	179.84 (5)	C3—C4—H4A	109.5
O1—Re1—O3^i^	89.75 (5)	C3—C4—H4B	109.5
O2^i^—Re1—O3^i^	90.13 (5)	H4A—C4—H4B	109.5
O1—Re1—O4	90.37 (5)	C3—C4—H4C	109.5
O2^i^—Re1—O4	89.75 (5)	H4A—C4—H4C	109.5
O3^i^—Re1—O4	179.87 (5)	H4B—C4—H4C	109.5
O1—Re1—Re1^i^	89.63 (4)	O4—C5—O3	120.86 (15)
O2^i^—Re1—Re1^i^	90.27 (4)	O4—C5—C6	118.98 (16)
O3^i^—Re1—Re1^i^	90.15 (4)	O3—C5—C6	120.16 (16)
O4—Re1—Re1^i^	89.80 (4)	C5—C6—C7	112.87 (16)
O1—Re1—Cl1	88.98 (4)	C5—C6—H6A	109.0
O2^i^—Re1—Cl1	91.13 (4)	C7—C6—H6A	109.0
O3^i^—Re1—Cl1	90.89 (4)	C5—C6—H6B	109.0
O4—Re1—Cl1	89.16 (4)	C7—C6—H6B	109.0
Re1^i^—Re1—Cl1	178.254 (11)	H6A—C6—H6B	107.8
C1—O1—Re1	120.09 (10)	C8—C7—C6	113.24 (19)
C1—O2—Re1^i^	119.38 (11)	C8—C7—H7A	108.9
C5—O3—Re1^i^	119.40 (11)	C6—C7—H7A	108.9
C5—O4—Re1	119.78 (11)	C8—C7—H7B	108.9
O1—C1—O2	120.63 (15)	C6—C7—H7B	108.9
O1—C1—C2	118.72 (15)	H7A—C7—H7B	107.7
O2—C1—C2	120.64 (15)	C7—C8—H8A	109.5
C1—C2—C3	115.01 (15)	C7—C8—H8B	109.5
C1—C2—H2A	108.5	H8A—C8—H8B	109.5
C3—C2—H2A	108.5	C7—C8—H8C	109.5
C1—C2—H2B	108.5	H8A—C8—H8C	109.5
C3—C2—H2B	108.5	H8B—C8—H8C	109.5
H2A—C2—H2B	107.5	N1—C9—C10	180.0 (3)
C4—C3—C2	113.00 (17)	C9—C10—H10A	109.5
C4—C3—H3A	109.0	C9—C10—H10B	109.5
C2—C3—H3A	109.0	H10A—C10—H10B	109.5
C4—C3—H3B	109.0	C9—C10—H10C	109.5
C2—C3—H3B	109.0	H10A—C10—H10C	109.5
H3A—C3—H3B	107.8	H10B—C10—H10C	109.5
			
Re1—O1—C1—O2	−1.0 (2)	Re1—O4—C5—O3	1.2 (2)
Re1—O1—C1—C2	178.70 (12)	Re1—O4—C5—C6	−178.97 (12)
Re1^i^—O2—C1—O1	0.8 (2)	Re1^i^—O3—C5—O4	−1.1 (2)
Re1^i^—O2—C1—C2	−178.84 (12)	Re1^i^—O3—C5—C6	179.11 (12)
O1—C1—C2—C3	167.38 (16)	O4—C5—C6—C7	−58.1 (2)
O2—C1—C2—C3	−12.9 (2)	O3—C5—C6—C7	121.71 (19)
C1—C2—C3—C4	−70.2 (2)	C5—C6—C7—C8	−67.9 (2)

Symmetry code: (i) −*x*, −*y*, −*z*+1.
